# MicroRNA-23a promotes pancreatic cancer metastasis by targeting epithelial splicing regulator protein 1

**DOI:** 10.18632/oncotarget.20692

**Published:** 2017-08-24

**Authors:** Guo Wu, Zhonghu Li, Peng Jiang, Xi Zhang, Yingqiang Xu, Kai Chen, Xiaowu Li

**Affiliations:** ^1^ Department of Hepatobiliary Surgery Institute, South Western Hospital, Third Military Medical University, Chongqing 400038, China

**Keywords:** microRNA-23a (miR-23a), pancreatic cancer, ESRP1, epithelial-mesenchymal transition (EMT), TGF-β1

## Abstract

miR-23a plays vital roles in various cancer metastases. Here, we found that miR-23a expression was significantly up-regulated in pancreatic cancer tissues compared with adjacent normal tissues. miR-23a up-regulation was significantly associated with differentiated degree, lymphoid nodal status, tumor invasion and poor survival rate in pancreatic cancer patients. We also found that miR-23a expression was significantly up-regulated in lymph node metastatic tissues and in pancreatic cancer cells that underwent epithelial-mesenchymal transition (EMT). miR-23a down-regulation blocked TGF-β1-induced EMT and reversed the phenotype of EMT in Panc-1 cells. Furthermore, miR-23a down-regulation inhibited Panc-1 cells migration and invasion *in vitro* and liver metastases *in vivo*. But the effect of miR-23a up-regulation in Aspc-1 cells was opposite to that of miR-23a down-regulation in Panc-1 cells. Epithelial splicing regulatory protein 1 (ESRP1) was identified as a direct target of miR-23a. Restoration of ESRP1 rescued the effect of miR-23a on pancreatic cancer cell progression. Moreover, miR-23a up-regulation in Aspc-1 cells induced a shift in CD44 expression from variant isoforms (CD44v) to the standard isoform (CD44s) together with increased FGFR2 IIIc mRNA levels, and decreased FGFR2 IIIb expression during EMT. But the effect of miR-23a down-regulation in Panc-1 cells was opposite to that of miR-23a up-regulation in Aspc-1 cells. In addition, the effect of miR-23a up-regulation was partly reversed by ESRP1 over-expression. Taken together, our findings indicated that miR-23a functions as an oncogene by regulating ESRP1 in pancreatic cancer.

## INTRODUCTION

Pancreatic cancer is one of the most malignant solid tumors in human and is characterized by its extremely aggressive nature, which involves local invasion and early metastasis [1]. Although an increasing number of therapies, including surgical resection, chemotherapy and radiotherapy, have been used in recent years, the overall 5-year survival rate is still less than 5% [2]. Metastasis, the process by which cancer cells spread from their primary site to other parts of the body, is responsible for approximately 90% of cancer-related deaths [3]. The epithelial-mesenchymal transition (EMT) is considered to be an initial and key step in the metastatic cascade [4]. During the EMT process, neoplastic cells become motile and invasive and leave the primary epithelial region, thereby contributing to the metastatic potential of carcinomas [5, 6]. Therefore, it is critical to understand the mechanisms by which EMT is regulated to facilitate the development of effective therapeutic strategies for the treatment of recurrent and metastatic cancer.

MicroRNAs (miRNAs) are small, non-coding RNA molecules, which post-transcriptionally regulate gene expression by directly binding to the 3'-untranslated region (3' UTR) of their target genes and inducing target mRNA degradation or suppressing target mRNA translation. Several microRNAs have been shown to either suppress EMT [7–11] or promote EMT and tumor metastasis [12–14]. Remarkably, miR-23a has been proposed to function as either an oncogene or a tumor suppressor in various human cancers. miR-23a promotes both colon carcinoma cell invasion and metastasis through inhibition of the MTSS gene [15] and the proliferation of liver cancer and gastric adenocarcinoma cells [16, 17]. In contrast, miR-23a expression was decreased and functioned as a tumor suppressor in osteosarcoma [18, 19]. Recently, miRNA microarray analysis showed that compared with normal pancreatic tissue, miR-23a was up-regulated in pancreatic ductal adenocarcinoma (PDAC) [20]. Moreover, miR-23a was up-regulated in pancreatic cancer cells with major elongation capacity, which appeared to be a significant process during PDAC peritoneal metastasis [21]. However, the role of miR-23a in pancreatic cancer development remains largely unknown. In the present study, we sought to investigate the potential role and mechanism of miR-23a in PDAC EMT and metastasis. Our results demonstrated that miR-23a promoted EMT programming and metastasis by directly targeting epithelial splicing regulatory protein 1 (ESRP1) and consequently regulating CD44 splicing, as well as FGFR2 IIIb and FGFR2 IIIc mRNA levels.

## RESULTS

### miR-23a was aberrantly up-regulated in pancreatic cancer tissues and correlated with poor tumor progression

**Figure 1:**
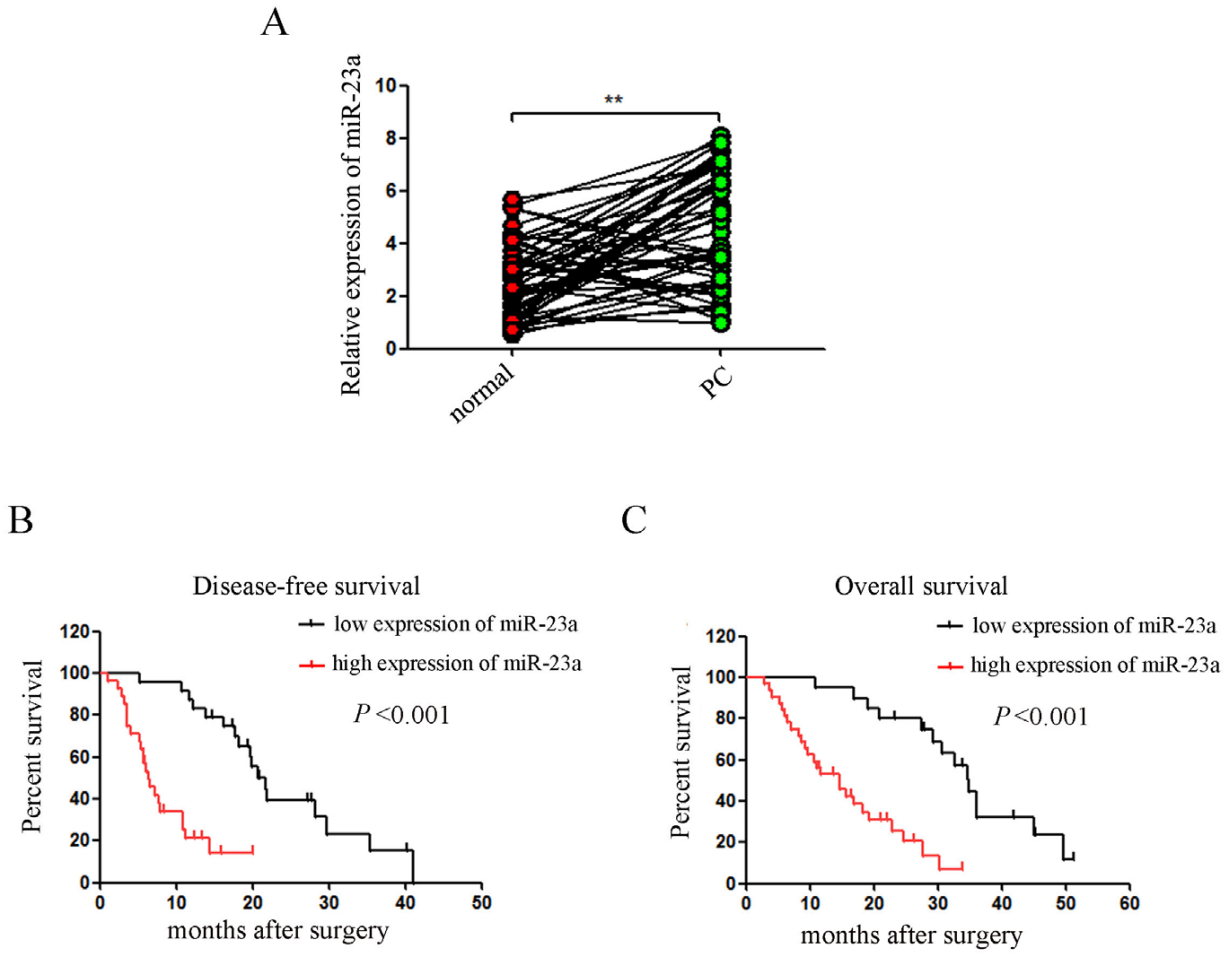
miR-23a expression was aberrantly up-regulated in pancreatic cancer tissues and correlated with disease progression **(A)** miR-23a expression was determined in 52 pancreatic tumor samples and their pair–matched adjacent normal samples by qRT-PCR. U6 was used as a loading control. **(B** and **C)** Kaplan–Meier curves for the survival time of patients with pancreatic cancer divided according to miR-23a expression. **, *P*<0.01.

To determine the expression of miR-23a in pancreatic cancer tissues, we analyzed miR-23a expression levels in 52 pancreatic tumor samples and their pair–matched adjacent normal samples by qRT-PCR. Our results showed that miR-23a expression was significantly up-regulated in pancreatic cancer tissues compared with adjacent normal tissues (Figure 1A; 4.69±2.07 *vs*. 2.48±1.53, *P*<0.01). Clinicopathological analyses of 52 pancreatic cancer patients showed that elevated miR-23a expression was significantly associated with differentiated degree (*P*<0.05), lymphoid nodal status (*P*<0.01) and tumor invasion (*P*<0.05) (Table 1). Moreover, Kaplan–Meier survival analysis showed that patients with higher miR-23a expression had significantly reduced disease-free survival and overall survival rates (all *P*< 0.001; Figure 1B and 1C).

**Table 1:** Correlations between miR-23a expression and clinicopathological characteristics of patients with pancreatic cancer

Clinical factor	miR-23a expression		*P* value
	Low expression (n=20)	High expression (n=32)	
Sex			
Male	9	14	0.930
Female	11	18	
Age(y)			
<60	10	19	0.508
≥60	10	13	
Tumor location			
Pancreatic head	14	22	0.924
Pancreatic body and tail	6	10	
Differentiated degree			
Poor differentiated	8	22	0.041
Well +moderate differentiated	12	10	
Lymphoid nodal(N) status			
Absent	15	12	0.008
Present	5	20	
Tumor (T) invasion			
T1 + T2	12	9	0.023
T3 + T4	8	23	
Distant metastasis(M) status			
M0	17	25	0.541
M1	3	7	

### miR-23a was up-regulated in pancreatic cancer cells that underwent EMT

**Figure 2:**
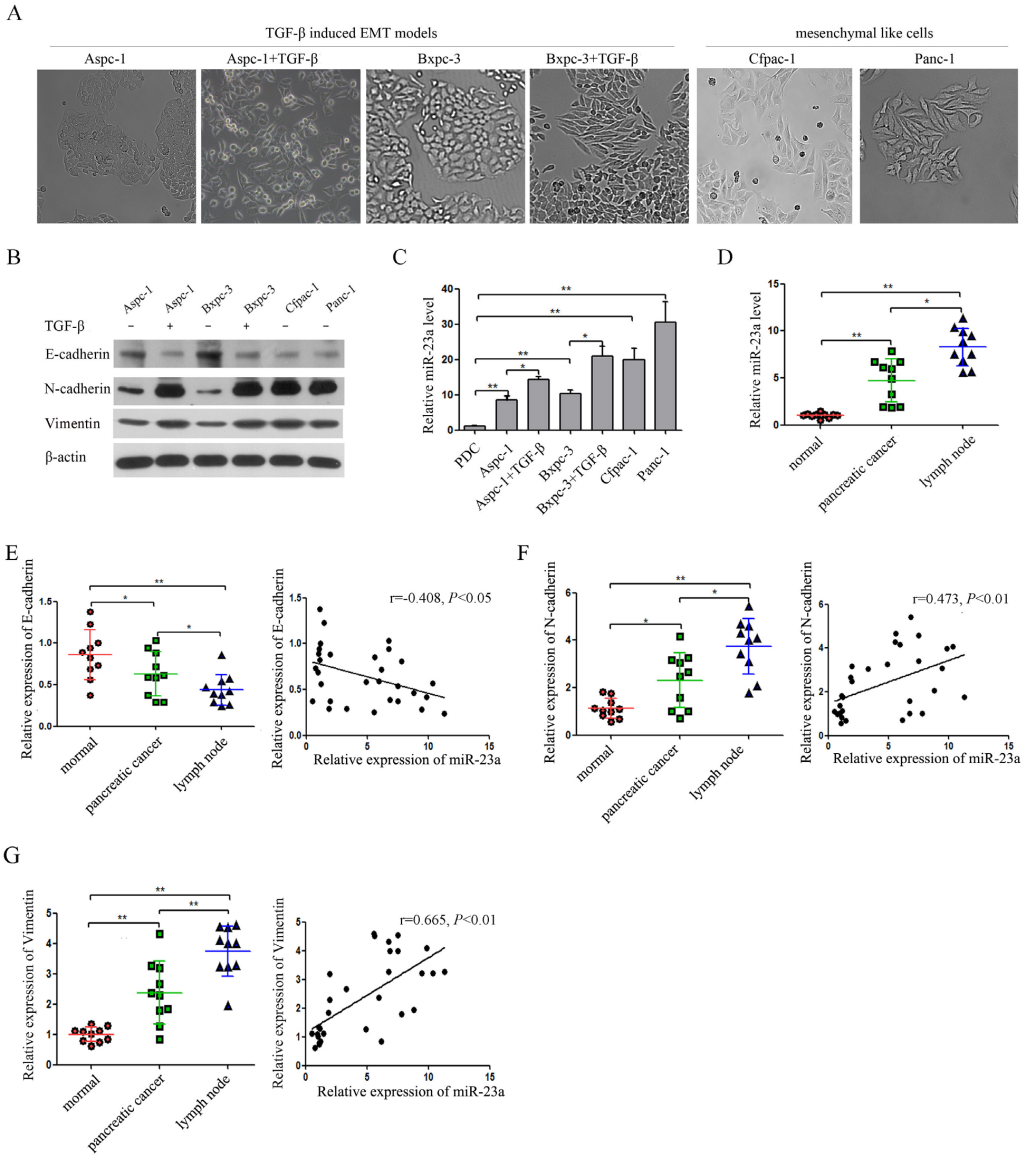
miR-23a expression was up-regulated in pancreatic cancer cells that underwent EMT and in lymph node metastatic tissues **(A)** Morphology analysis of Aspc-1 cells, Bxpc-3 cells, Cfpac-1 cells, Panc-1 cells, TGF-β1-treated Aspc-1 cells and TGF-β1-treated Bxpc-3 cells. **(B** and **C)** Analysis of E-cadherin, N-cadherin, Vimentin and miR-23a expression levels in Aspc-1 cells, Bxpc-3 cells, Cfpac-1 cells, Panc-1 cells, TGF-β1-treated Aspc-1 cells and TGF-β1-treated Bxpc-3 cells. **(D)** Analysis of miR-23a expression levels in human pancreatic cancer tissues, their adjacent normal tissues (normal) and lymph node metastatic tissues (lymph node). U6 was used as a loading control. **(E)** Expression of E-cadherin mRNA in human pancreatic cancer tissues, their adjacent normal tissues (normal) and lymph node metastases (lymph node). Data are shown as the mean ± SD (n=10). And the Spearman’s Correlation analysis clearly showed negative correlation between miR-23a and E-cadherin in tumor samples from pancreatic cancer patients. **(F** and **G)** Expression of N-cadherin and Vimentin mRNA in human pancreatic cancer tissues, their adjacent normal tissues (normal) and lymph node metastases (lymph node). Data are shown as the mean ± SD (n=10). And the Spearman’s Correlation analysis clearly showed positive correlation between miR-23a and E-cadherin mRNA expression (r=0.473, *P*<0.01) and Vimentin mRNA expression (r=0.665, *P*<0.01) in tumor samples from pancreatic cancer patients. *, *P*<0.05; **, *P*<0.01.

Aspc-1 and Bxpc-3 cells exhibited characteristic epithelial cobblestone-like morphology with high expression of the epithelial marker (E-cadherin) and low expression of the mesenchymal markers (N-cadherin and Vimentin) (Figure 2A and 2B). In contrast, Cfpac-1 and Panc-1 cells had a more elongated, fibroblast-like mesenchymal appearance with reduced E-cadherin expression and increased N-cadherin and Vimentin expression (Figure 2A and 2B). In addition, Aspc-1 and Bxpc-3 cells treated with TGF-β1 lost their epithelial morphology and acquired mesenchymal traits with reduced E-cadherin expression and increased N-cadherin and Vimentin expression (Figure 2A and 2B). Moreover, qRT-PCR analysis showed that miR-23a expression was significantly increased in pancreatic cancer cell lines compared with PDCs (Figure 2C). We also found that miR-23a expression was significantly up-regulated in Cfpac-1 and Panc-1 cells compared with Aspc-1 and Bxpc-3 cells (Figure 2C). Furthermore, miR-23a was markedly up-regulated in the cells that underwent EMT (Figure 2C). To further assess the correlation of miR-23a with pancreatic cancer, we analysed miR-23a expression in tumor tissues. Compared with primary cancer tissues and their adjacent normal pancreatic tissues, miR-23a was markedly up-regulated in lymph node metastatic tissues (Figure 2D). To further confirm the correlation of miR-23a with EMT, we analyzed the expression of E-cadherin, N-cadherin and Vimentin by qRT-PCR in the aforementioned tissues. Our results showed that E-cadherin mRNA was significantly decreased in pancreatic cancer tissues and lymph node metastases when compared with their adjacent normal pancreatic tissues, and inversely correlated with miR-23a expression (r=-0.408, *P*<0.05, Pearson correlation, Figure 2E). However, both N-cadherin and Vimentin mRNA were significantly increased in pancreatic cancer tissues and lymph node metastases when compared with their adjacent normal pancreatic tissues, and positively correlated with miR-23a expression (r=0.473, *P*<0.01; r=0.665, *P*<0.01; Pearson correlation, Figure 2F and 2G).

### miR-23a down-regulation suppressed TGF-β1 induced EMT

**Figure 3:**
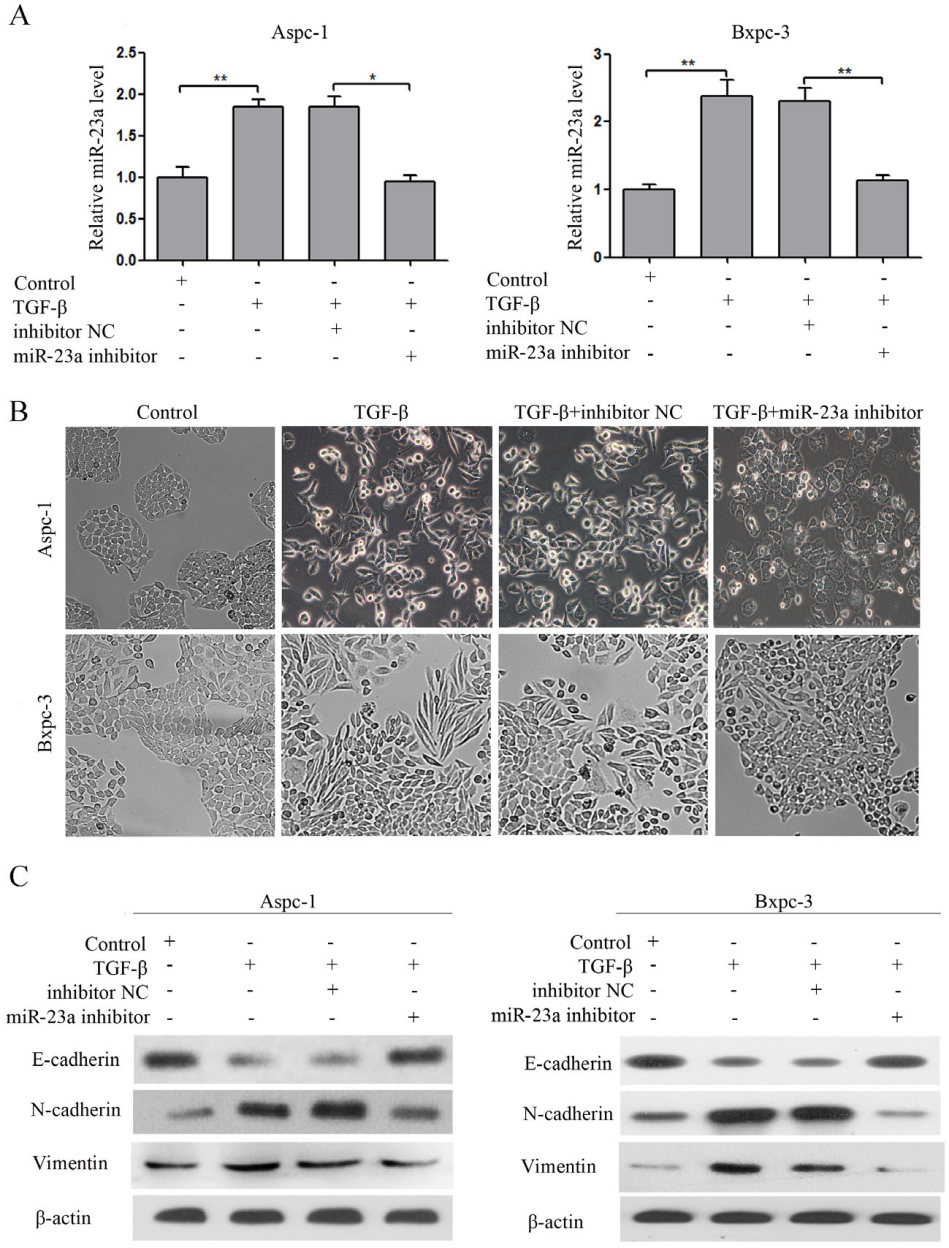
miR-23a down-regulation blocked TGF-β1 induced EMT **(A)** miR-23a expression levels were detected by qRT-PCR, and U6 was used as a loading control. **(B)** Morphology analysis of Aspc-1 and Bxpc-3 cells either treated with TGF-β1 alone or co-incubated with miR-23a inhibitors. **(C)** Western blot analysis of E-cadherin, N-cadherin and Vimentin protein expression in Aspc-1 and Bxpc-3 cells following the aforementioned treatments.

To evaluate the effect of miR-23a on pancreatic cancer cell EMT, we transfected miR-23a inhibitors (miR-23a inhibitor) and the inhibitor control (inhibitor NC) into Aspc-1 and Bxpc-3 cells treated with TGF-β1. The miR-23a expression in transfected cells was reversed (Figure 3A). miR-23a down-regulation blocked TGF-β1-induced EMT in Aspc-1 and Bxpc-3 cells (Figure 3B and 3C). The cobblestone-like appearance and E-cadherin expression remained intact, and N-cadherin and Vimentin expression were attenuated in the cells treated with TGF-β1 in combination with miR-23a inhibitors (Figure 3B and 3C).

### miR-23a was required for EMT

**Figure 4:**
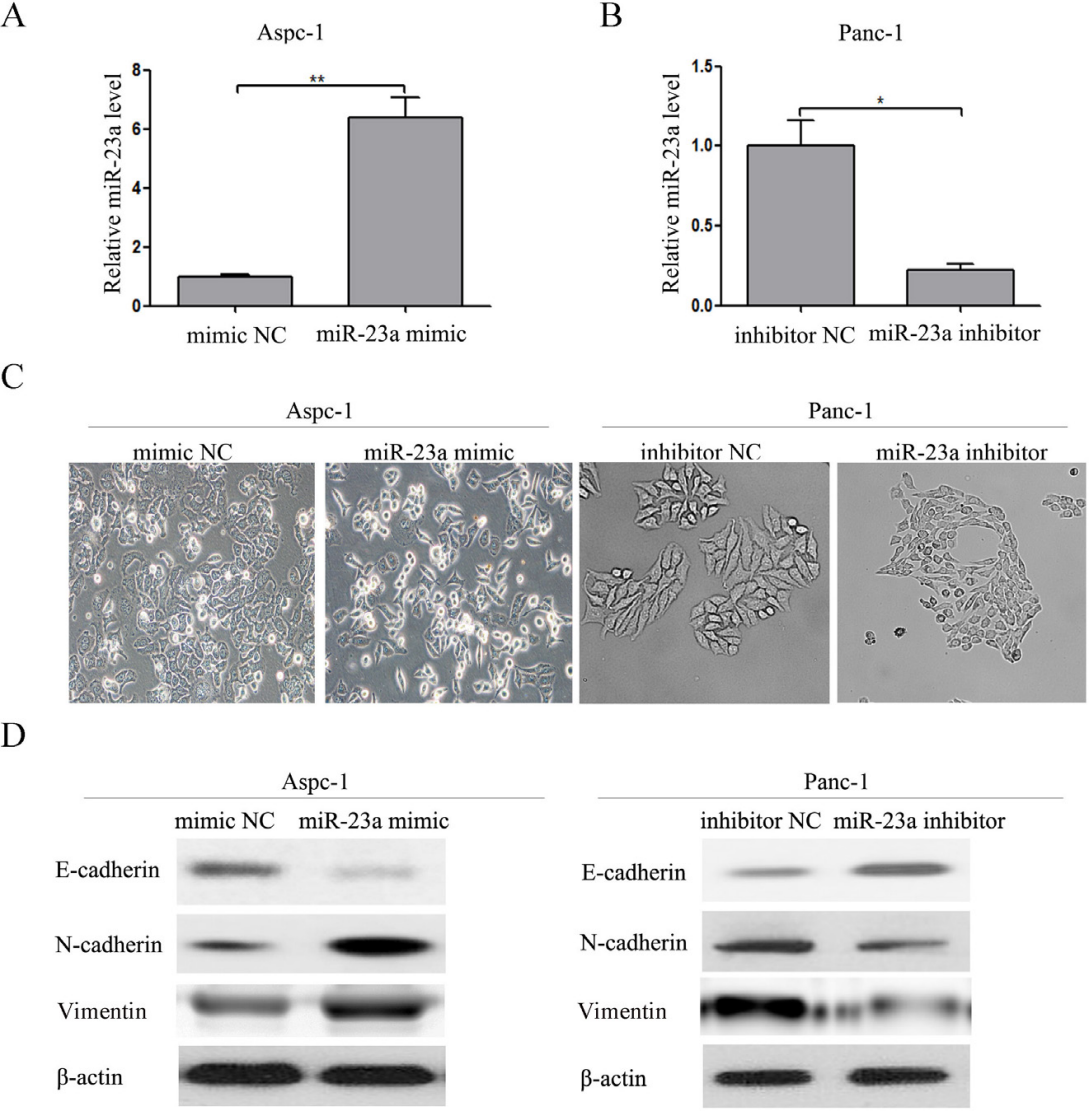
Up-regulation of miR-23a was required for EMT initiation and maintenance **(A)** Aspc-1 cells were transfected with miR-23a mimics or mimic NC, and the miR-23a expression levels were detected by qRT-PCR. U6 was used as a loading control. **(B)** Panc-1 cells were transfected with miR-23a inhibitors or inhibitor NC for 48 h, and the miR-23a expression levels were detected by qRT-PCR. U6 was used as a loading control. **(C)** Morphology analysis of Aspc-1 cells transfected with miR-23a mimics or mimic NC. And morphology analysis of Panc-1 cells transfected with miR-23a inhibitors or inhibitor NC. **(D)** Western blot analysis of E-cadherin, N-cadherin and Vimentin protein expression in Aspc-1 and Panc-1 cells following the aforementioned treatments.

Subsequently, we investigated whether overexpression of miR-23a had an effect on EMT and if miR-23a was required for mesenchymal cancer cells to maintain a mesenchymal phenotype. Firstly, we transfected miR-23a mimics and mimic NC into the Aspc-1 cells. The miR-23a expression in the transfected cells was significantly increased (Figure 4A). Compared with the mimic NC group, Aspc-1 cells transfected with miR-23a mimics showed an obvious shift in morphology from cobblestone-like to more spindle-shaped (Figure 4C). This morphological change was associated with increased N-cadherin and Vimentin expression and reduced E-cadherin expression in cells transfected with miR-23a mimics (Figure 4D). Secondly, we transfected miR-23a inhibitors and inhibitor NC into Panc-1 cells. The miR-23a expression in transfected cells was significantly decreased (Figure 4B). Compared with the inhibitor NC group, Panc-1 cells transfected with miR-23a inhibitors began to adopt an epithelia-like morphology (Figure 4C), associated with increased E-cadherin expression and reduced N-cadherin and Vimentin expression (Figure 4D).

### miR-23a promoted pancreatic cancer cell invasion and migration *in vitro* and metastasis *in vivo*

**Figure 5:**
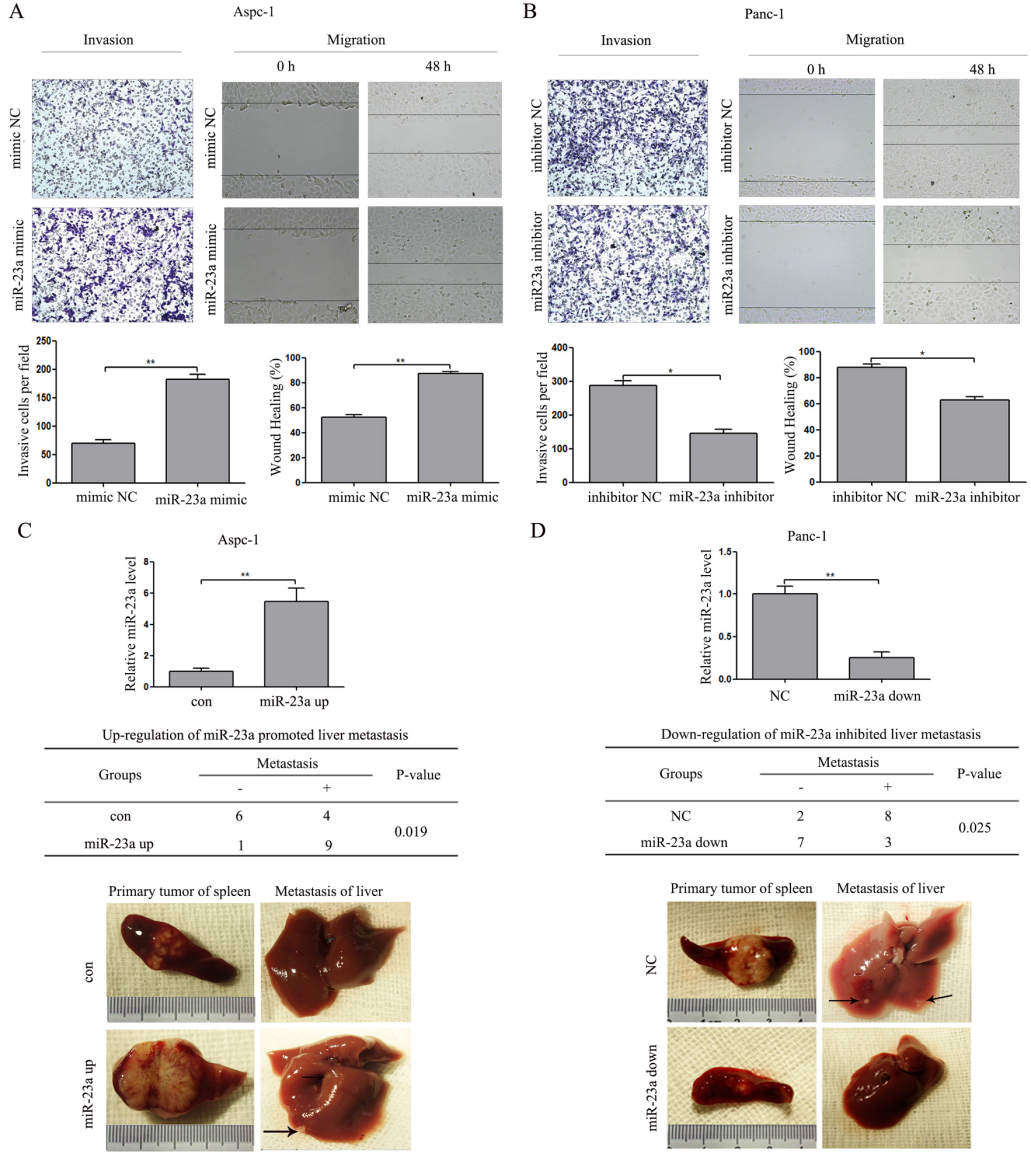
miR-23a induced PC cell invasion and migration *in vitro* and metastasis *in vivo* **(A)** Transwell invasion and wound healing assays using Aspc-1 cells transfected with miR-23a mimics or mimic NC. **(B)** Transwell invasion and wound healing assays using Panc-1 cells transfected with miR-23a inhibitors or inhibitor NC. **(C)** miR-23 over-expression in Aspc-1 cells by miR-23a up-regulation lentivirus inhibited pancreatic cancer cell growth and metastasis *in vivo*. Data are shown as the mean ± SD (n=10). **(D)** The inhibition of miR-23 expression in Panc-1 cells by miR-23a down-regulation lentivirus repressed pancreatic cancer cell growth and metastasis *in vivo*. Data are shown as the mean ± SD (n=10). *, *P*<0.05; **, *P*<0.01.

To determine whether miR-23a regulates invasion and migration in pancreatic cancer cells, we performed *in vitro* gain-of-function assays using miR-23a mimics in epithelial phenotype pancreatic cancer cells (Aspc-1) and loss-of-function assays using miR-23a inhibitors in mesenchymal phenotype pancreatic cancer cells (Panc-1). Transwell assays and wound healing assays showed that miR-23a over-expression significantly promoted Aspc-1 cell invasion and migration (Figure 5A). In contrast, miR-23a down-regulation significantly repressed Panc-1 cell invasion and migration (Figure 5B). To confirm the effect of miR-23a on metastasis *in vivo*, we established stable Aspc-1 cell line overexpressing miR-23a (miR-23a up) and stable Panc-1 cell line inhibiting miR-23a (miR-23a down). Then, we inoculated the cells into the splenic capsules of nude mice. The primary splenic tumours and liver metastases were inspected after 6 weeks. As shown in Figure 5C, the diameters of the primary splenic tumours in the miR-23a over-expression group mice were significantly increased compared with that in con group mice (Figure 5C; 1.88±0.48 *vs*. 0.90±0.27, *P*<0.01), and the number of mice with liver metastases in the miR-23a over-expression group was also significantly increased compared with that in con group (Figure 5C; 9/10 *vs*. 4/10, *P*<0.05). However, the diameters of the primary splenic tumours in the miR-23a down-regulation group mice were significantly decreased compared with that in NC group mice (Figure 5D; 0.64±0.29 *vs*. 1.52±0.26, *P*<0.01), and the number of mice with liver metastases in the miR-23a down-regulation group was also significantly decreased compared with that in NC group (Figure 5D; 3/10 *vs*. 8/10, *P*<0.05). Our results strongly indicate that miR-23a could promote pancreatic cancer cell invasion, migration and metastasis.

### miR-23a down-regulation induced global expression changes of genes related to proliferation and progression

**Figure 6:**
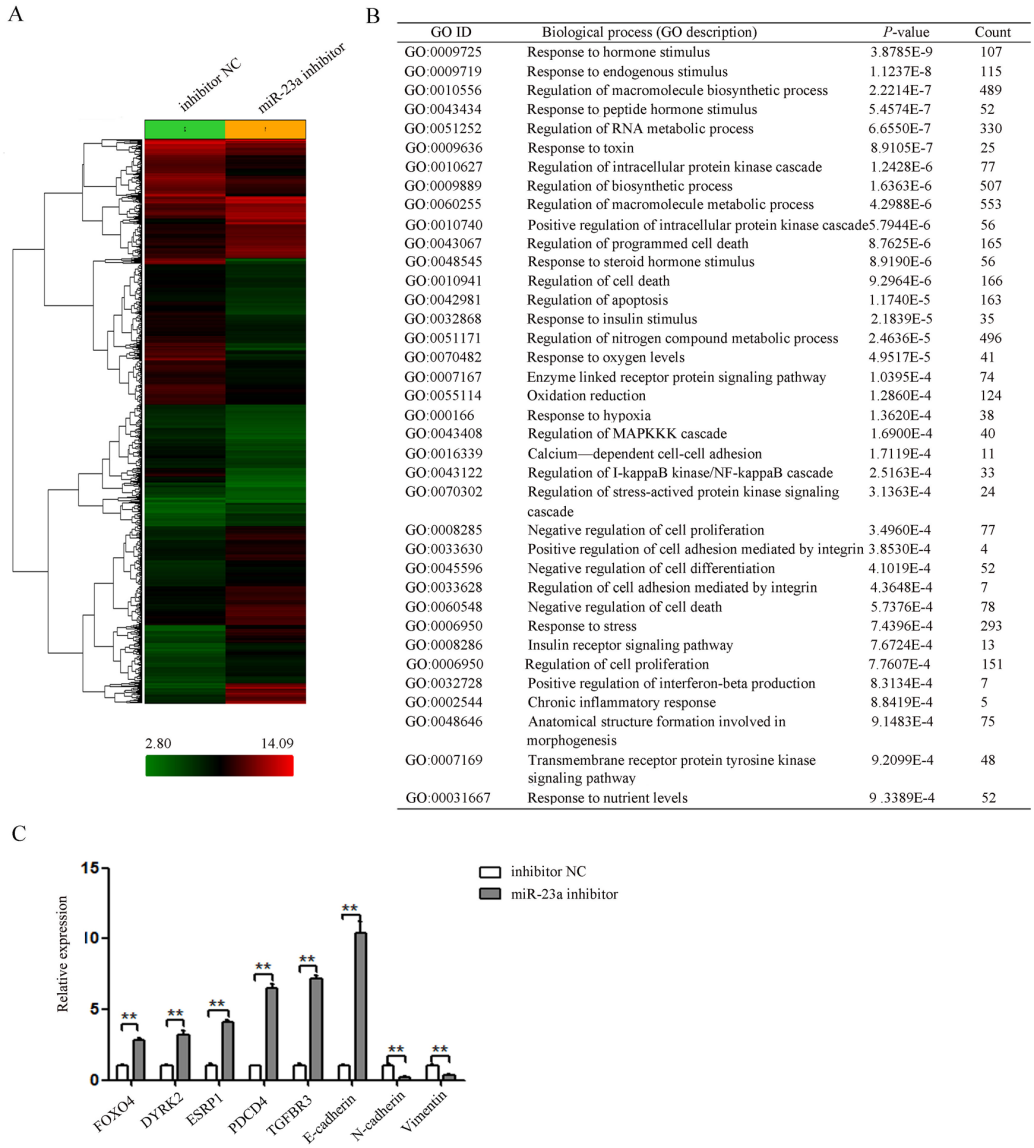
miR-23a down-regulation repressed a subset of gene involved proliferation and progression **(A)** Clustering of differentially expressed genes between the miR-23a inhibitor group and the inhibitor NC group. **(B)** Gene ontology analysis of up- and down-regulated genes related to proliferation and progression (miR-23a inhibitor group vs. the inhibitor NC group). **(C)** The mRNA levels of selected candidate genes related to EMT and metastasis were determined by qRT-PCR. **, *P*<0.01.

To determine which gene was affected by miR-23a in pancreatic cancer, the global gene expression changes induced by miR-23a down-regulation were determined by comparing the gene expression profiles between the miR-23a inhibitor group and the inhibitor NC group Pan-1 cells using the Affymetrix Gene Chip Human Gene 1.0 ST Array. Of the 29,000 genes analysed, 2681 genes showed statistically significant differences in gene expression based on a cut-off value of >2-fold differential expression by miR-23a down-regulation (Figure 6A).

The genes with significant expression changes were submitted to the Biological Networks Gene Ontology tool (BiNGO) for Gene Ontology (GO) analysis. According to GO terms, these genes were divided into three major categories: biological process, molecular function, and cellular component. The GO terms representing biological processes related to proliferation and progression were listed in Figure 6B. And the GO terms representing molecular function and cellular compartment were listed in Supplementary Table 2.

To validate the microarray results, the expression of 8 genes related to EMT in cancer was determined by qRT-PCR (Figure 6C). Our results confirmed the expression changes of 7 genes (FOXO4, DYRK2, ESRP1, PDCD4, TGFBR3, E-cadherin, N-cadherin) identified by microarray. However, Vimentin without significant expression change based on microarray results was found to be significantly suppressed in cells after transfection with miR-23a inhibitor by qRT-PCR (Figure 6C). These results suggested that the sensitivity of microarray was lower than that of qRT-PCR.

### miR-23a directly targeted ESRP1 through binding to its 3' UTR

**Figure 7:**
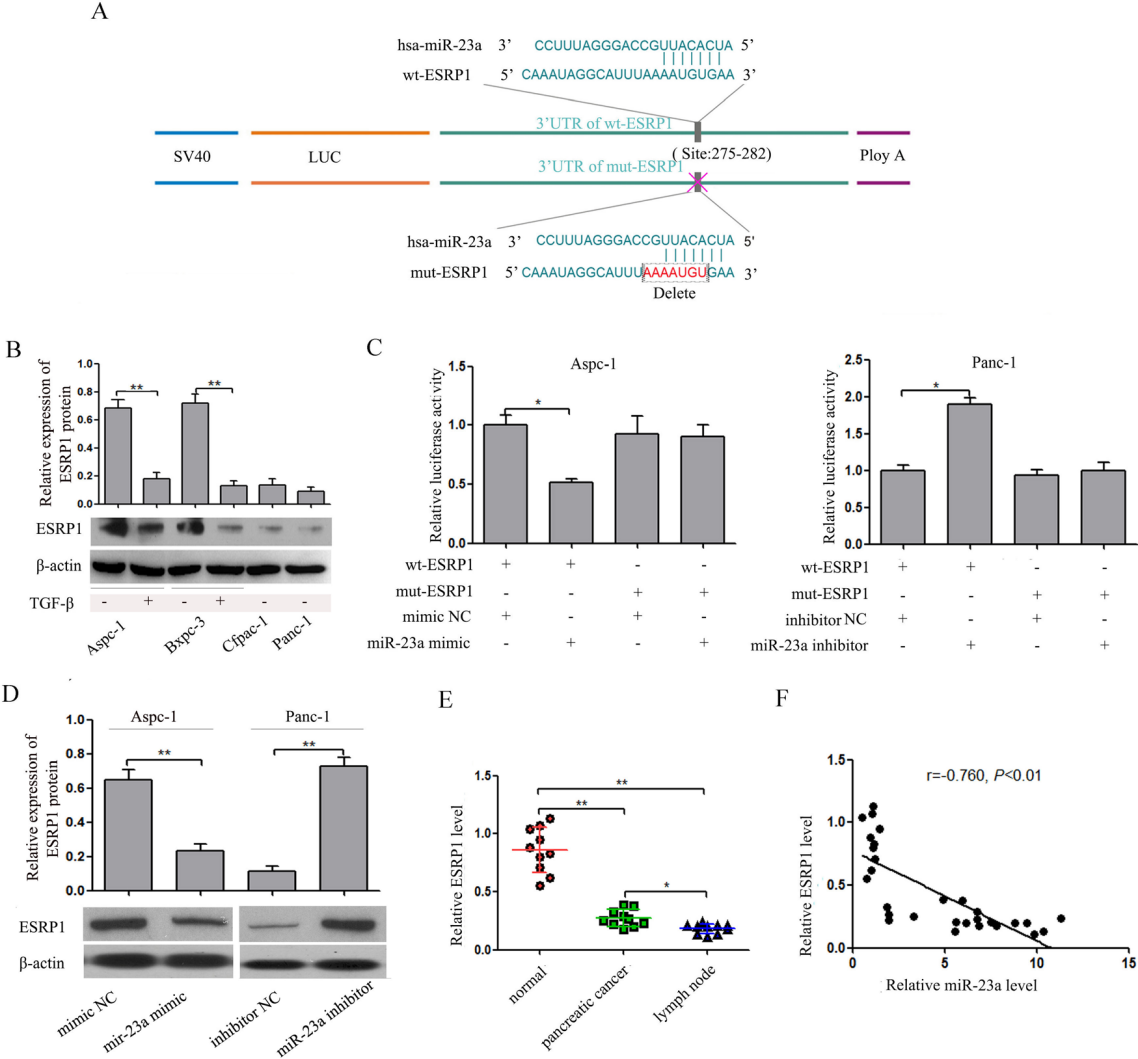
miR-23a down-regulated ESRP1 by binding to its 3' UTR **(A)** miR-23a and its putative binding sequences in the ESRP1 3' UTR. Mutations were generated in the sequence complementary to the miR-23a target binding region. **(B)** Analysis of ESRP1 expression levels in Aspc-1 cells, Bxpc-3 cells, Cfpac-1 cells, Panc-1 cells, TGF-β1-treated Aspc-1 cells and TGF-β1-treated Bxpc-3 cells. **(C)** In Aspc-1 cells, miR-23a over-expression suppressed the luciferase activity of the wild-type ESRP1 3' UTR (wt-ESRP1) but not the mutant. Data are shown as the mean ± SD (n=3). In Panc-1 cells, miR-23a inhibition augmented the activity of firefly luciferase reporters containing the wt-ESRP1 but not the mutant. Data are shown as the mean ± SD (n=3). **(D)** Western blot analysis of ESRP1 expression in Aspc-1 cells transfected miR-23a mimics and in Panc-1 cells transfected miR-23a inhibitor. **(E)** Expression levels of ESRP1 mRNA in human pancreatic cancer tissues, their adjacent normal tissues (normal) and their lymph node metastases (lymph node). Data are shown as the mean ± SD (n=10). **(F)** The Spearman’s Correlation analysis clearly showed negative correlation between miR-23a and ESRP1 mRNA expression in tumor samples from pancreatic cancer patients. *, *P*<0.05; **, *P*<0.01.

It is well known that miRNAs participate in various physiological and pathological processes by directly regulating target gene expression. Putative miR-23a targets genes involved in EMT and metastasis were predicted by comparing our list of putative downstream genes of miR-23a with the list of direct functional targets of miR-23a as determined by bioinformatics search using Targetscan (www.targetscan.org). Finally, ESRP1 was identified as a direct target of miR-23a. In the 3' UTR of ESRP1, one binding site of miR-23a was predicted by TargetScan (Figure 7A). We found that ESRP1 expression was significantly down-regulated in Cfpac-1 and Panc-1 cells compared with Aspc-1 and Bxpc-3 cells (Figure 7B). Furthermore, ESRP1 expression was markedly down-regulated in the cells that underwent EMT (Figure 7B). To examine whether miR-23a directly interacts with the 3'UTR of ESRP1, we used the dual luciferase reporter assay. When Aspc-1 cells were co-transfected with wt-ESRP1 3' UTR and either miR-23a mimic or mimic NC, luciferase activity was significantly reduced compared with the transfected control (Figure 7C). In addition, the miR-23a-mediated repression of luciferase activity was abolished by the mutant putative binding site (Figure 7C). In contrast, when Panc-1 cells were co-transfected with wt-ESRP1 3' UTR and either miR-23a inhibitors or inhibitor NC, luciferase activity was significantly increased. Moreover, the miR-23a-mediated enhancement of luciferase activity was abolished by the mutant putative binding site (Figure 7C). We also found that ESRP1 expression was decreased in Aspc-1 cells treated with miR-23a mimics, while ESRP1 expression was increased in Panc-1 cells treated with miR-23a inhibitors (Figure 7D). Furthermore, we examined the expression of ESRP1 mRNA in the three types of tissues and found that ESRP1 mRNA was significantly decreased in pancreatic cancer tissues and lymph node metastases (Figure 7E) and inversely correlated with miR-23a expression (Figure 7F). These results indicated that miR-23a directly targeted ESRP1.

### ESRP1 restoration partially reversed the effect of miR-23a on pancreatic cancer cell EMT

**Figure 8:**
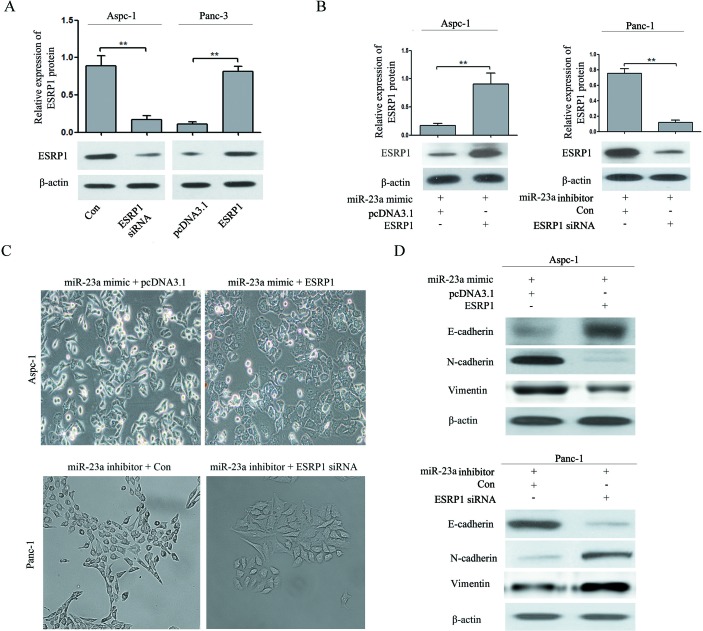
ESRP1 restoration partially reversed the effect of miR-23a on pancreatic cancer cell EMT **(A)** Western blot analysis of ESRP1 in Aspc-1 cells transfected with either ESRP1 siRNA or negative control siRNA (Con) and in Panc-1 cells transfected with either an ESRP1 over-expression (ESRP1) vector or control plasmid (pcDNA3.1). **(B)** Western blot analysis of ESRP1 in Aspc-1 cells co-transfected with miR-23a mimics and either pcDNA3.1 or the ESRP1 over-expression vector and in Panc-1 cells co-transfected with miR-23a inhibitors and either negative control siRNA (Con) or ESRP1 siRNA. **(C)** Morphology analysis of Aspc-1 cells co-transfected with miR-23a mimic and either pcDNA3.1 or the ESRP1 over-expression vector. And morphology analysis of Panc-1 cells co-transfected with miR-23a inhibitors and either negative control siRNA (Con) or ESRP1 siRNA. **(D)** Western blot analysis of E-cadherin, N-cadherin and Vimentin protein expression in Aspc-1 and Panc-1 cells following the aforementioned treatments.

To further determine the role of ESRP1 in the miR-23a-mediated effect on pancreatic cancer cell EMT, rescue experiments were performed. We constructed ESRP1 siRNA and an ESRP1 over-expression vector. Western blot analysis showed that ESRP1 siRNA significantly reduced ESRP1 protein expression in Aspc-1 cells, and the ESRP1 over-expression vector significantly increased ESRP1 protein expression in Panc-1 cells (Figure 8A). Then, we up-regulated ESRP1 expression in Aspc-1 cells after transfecting miR-23a mimics and down-regulated ESRP1 expression in Panc-1 cells after transfecting miR-23a inhibitors. Western blot analysis showed that in Aspc-1 cells pretreated with miR-23a mimics, the ESRP1 over-expression vector restored ESRP1 protein expression compared with cells transfected with pcDNA3.1, and in Panc-1 cells pretreated with miR-23a inhibitors, ESRP1 siRNA reduced ESRP1 protein expression compared with the control group (Figure 8B). ESRP1 up-regulation reversed the EMT-promoting effects of miR-23a mimics in Aspc-1 cells, leading to morphological and molecular changes consistent with mesenchymal-epithelial transition (MET) (Figure 8C and 8D). ESRP1 down-regulation rescued the repression of Panc-1 cells EMT due to miR-23a inhibitors, leading to morphological and molecular changes consistent with EMT (Figure 8C and 8D).

### ESRP1 restoration partially reversed the effect of miR-23a on pancreatic cancer cell invasion and migration

**Figure 9:**
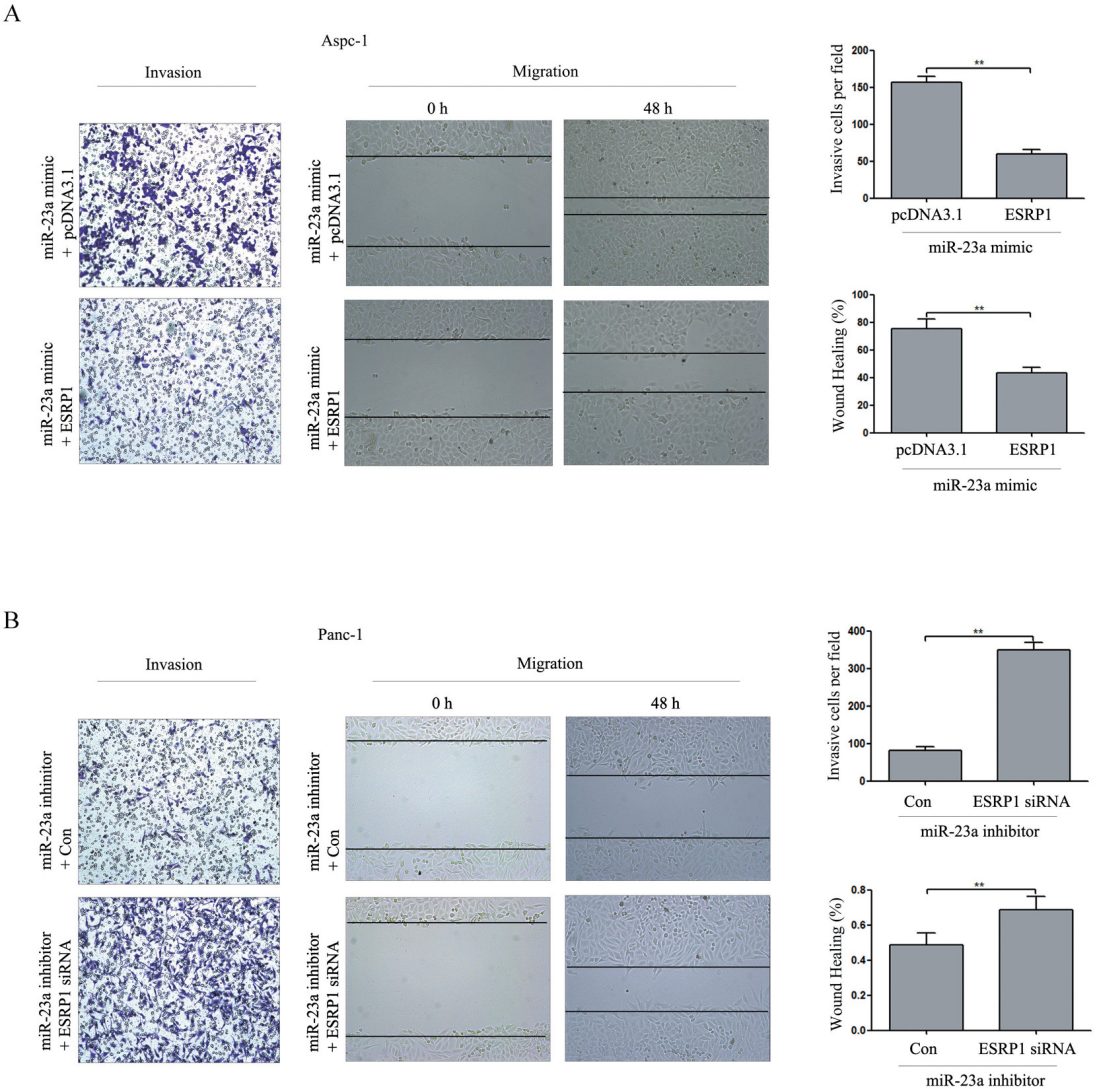
ESRP1 restoration partially reversed the effect of miR-23a on PC cell invasion and migration *in vitro* **(A)** Aspc-1 cells were co-transfected with miR-23a mimics and either pcDNA3.1 or the ESRP1 over-expression vector for 48 h, and then cell invasion and wound healing was detected. Data are shown as the mean ± SD (n=3). **(B)** Panc-1 cells were co-transfected with miR-23a inhibitors and either negative control siRNA (Con) or ESRP1 siRNA for 48 h, and then cell invasion and wound healing was detected. Data are shown as the mean ± SD (n=3). **, *P*<0.01.

As shown in Figure 9A, ESRP1 up-regulation reversed the promotion of invasion and migration caused by miR-23a mimics. Furthermore, ESRP1 down-regulation rescued the inhibition of invasion and migration caused by miR-23a inhibitors (Figure 9B). These data suggested that the effects of miR-23a on pancreatic cancer cell invasion and migration were achieved by directly targeting ESRP1.

### miR-23a repressed the expression of ESRP1 and consequently affected CD44 splicing, as well as FGFR2 IIIb and FGFR2 IIIc mRNA levels in pancreatic cancer cells

**Figure 10:**
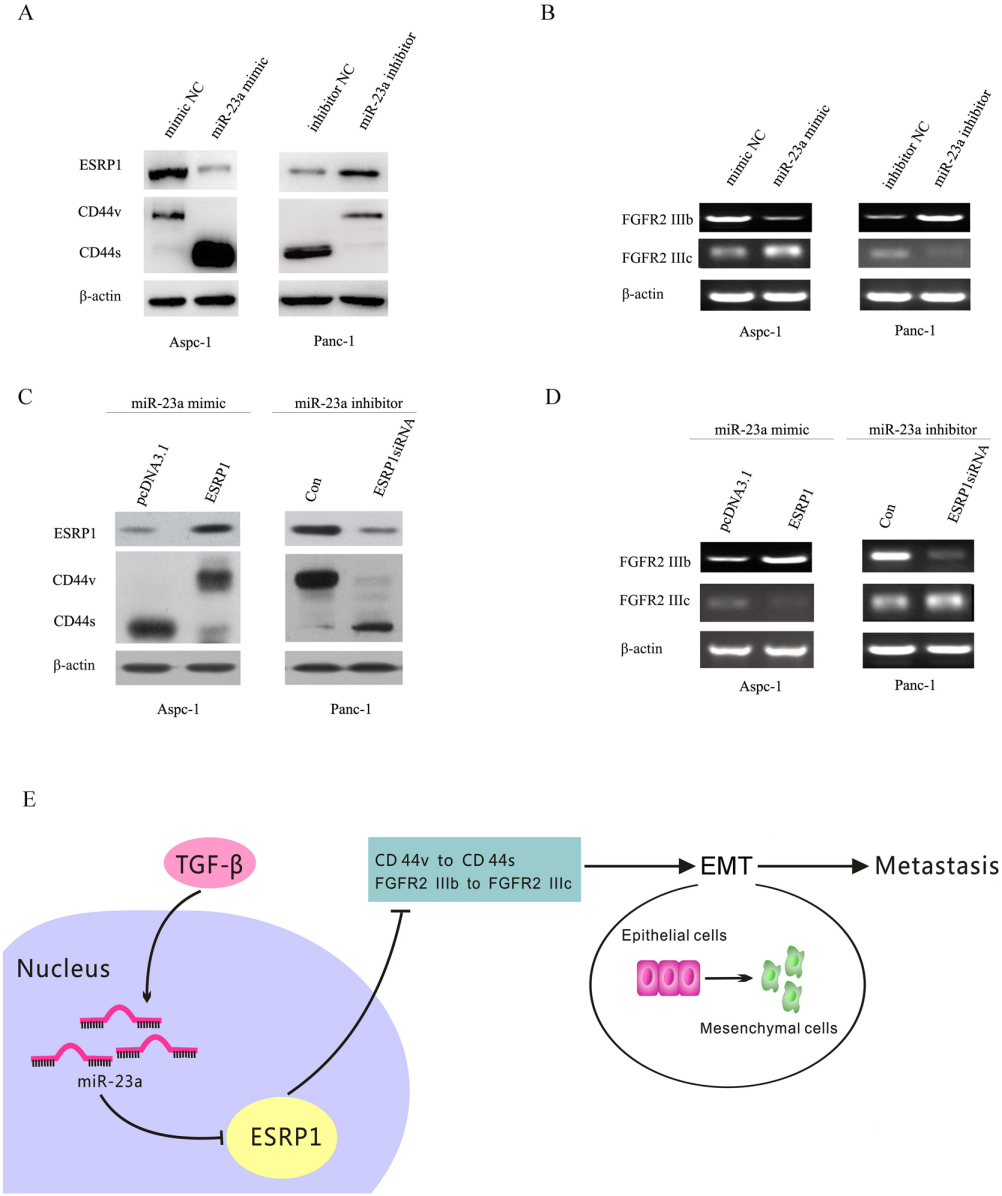
miR-23a regulated CD44 splicing as well as FGFR2 IIIb and FGFR2 IIIc mRNA levels by targeting ESRP1 **(A)** Western blot analysis of ESRP1 and the CD44 isoforms in Aspc-1 cells transfected with either mimic NC or miR-23a mimics and in Panc-1 cells transfected with either inhibitor NC or miR-23a inhibitors. **(B)** RT-PCR analysis of FGFR2 IIIb and FGFR2 IIIc mRNA levels in Aspc-1 cells transfected with either mimic NC or miR-23a mimics and in Panc-1 cells transfected with either inhibitor NC or miR-23a inhibitors. **(C)** The effects of ESRP1 restoration on the expression of ESRP1 and switching between CD44 isoforms expressions were detected by Western blot. **(D)** The effects of ESRP1 restoration on the FGFR2 IIIb and FGFR2 IIIc mRNA expression levels were detected by RT-PCR. **(E)** Abridged general view of the regulatory relationships in the miR-23a –ESRP1/CD44 signaling pathway. miR-23a up-regulation promotes pancreatic cancer EMT and metastasis by inhibiting ESRP1. ESRP1 down-regulation induces CD44 splice isoform switching as well as the changes of FGFR2 IIIb and FGFR2 IIIc mRNA levels.

Previous studies have shown that ESRP1 down-regulation was correlated with EMT as well as with changes in the FGFR2, CD44, CTNND1 (p120-Catenin) and ENAH transcripts [23]. In breast and pancreatic cancer, ESRP1 down-regulation promoted synthesis of CD44s isoform [24]. Changes in the expressed CD44 isoforms have been suggested to be associated with EMT [25]. In addition, ESRP1 regulates the expression pattern of FGFR-2 isoforms; attenuates cell growth, migration, invasion, and metastasis and is a favourable prognostic factor in PDAC [26]. As shown in Figure 10A, miR-23a up-regulation significantly reduced ESRP1 expression and induced a switch from CD44v to CD44s in Aspc-1 cells, but miR-23a down-regulation significantly induced a switch from CD44s to CD44v in Panc-1 cells. Moreover, miR-23a up-regulation significantly decreased FGFR2 IIIb mRNA levels and increased FGFR2 IIIc mRNA levels in Aspc-1 cells, but miR-23a down-regulation significantly increased FGFR2 IIIb mRNA levels and decreased FGFR2 IIIc mRNA levels in Panc-1 cells (Figure 10B).

To confirm the roles of ESRP1 in miR-23a regulated changes of CD44 isoform expression and the FGFR2 IIIb and FGFR2 IIIc mRNA levels, we up-regulated ESRP1 expression in Aspc-1 cells after transfecting miR-23a mimics and down-regulated ESRP1 expression in Panc-1 cells after transfecting miR-23a inhibitors. Our results showed that ESRP1 up-regulation in Aspc-1 cells reversed the miR-23a mimic-induced repression of ESRP1 and FGFR2 IIIb expression, the increase in FGFR2 IIIc expression, and the shift of CD44 isoform expression from CD44v to CD44s (Figure 10C and 10D). However, ESRP1 down-regulation in Panc-1 cells reversed the miR-23a inhibitor-induced increase in ESRP1 and FGFR2 IIIb expression, the repression of FGFR2 IIIc, and the changes of CD44 isoform expression from CD44s to CD44v (Figure 10C and 10D). Taken together, miR-23a may affect pancreatic cancer cell EMT and metastasis via regulating ESRP1 and its downstream factors (Figure 10E).

## DISCUSSION

It has been shown that miR-23a was up-regulated in pancreatic cancer cells with major elongation capacity, which appeared to be a significant process during PDAC peritoneal metastasis [21]. In the present study, we found that miR-23a expression was aberrantly up-regulated in pancreatic cancer tissues compared with adjacent normal tissues, associated with differentiated degree, lymphoid nodal status and tumor invasion. Moreover, patients with higher miR-23a expression levels had significantly reduced disease-free survival and overall survival rates. And miR-23a expression was significantly up-regulated both in cells with a natural mesenchymal phenotype (Cfpac-1 and Panc-1) and epithelial phenotype cells (Aspc-1 and Bxpc-3) by exposure to TGF-β1. In addition, miR-23a was markedly up-regulated in lymph node metastatic tissues compared with primary cancer tissues and their adjacent normal pancreatic tissues. Furthermore, a statistically negative correlation between the expression of miR-23a and the E-cadherin mRNA level was observed in pancreatic cancer tissues. And our results also showed that N-cadherin and Vimentin mRNA levels were positively correlated with miR-23a expression in pancreatic cancer tissues. Therefore, we concluded that miR-23a was able to promote EMT and the metastasis of pancreatic cancer cells. Our observations supported this conclusion. miR-23a down-regulation could prevent TGF-β1-induced EMT, leading to morphological and molecular changes consistent with MET. In addition, miR-23a up-regulation in epithelial phenotype cells (Aspc-1) induced EMT and increased cell invasion and migration *in vitro* and metastasis *in vivo*. However, miR-23a down-regulation in mesenchymal phenotype cells (Panc-1) leaded to opposite results. Moreover, DNA microarray results also showed that miR-23a down-regulation highlighted the expression of genes related to EMT, proliferation and progression in Panc-1 cells. These findings showed that miR-23a is required for EMT initiation and maintenance. Interestingly, miR-23a up-regulation induced an EMT-mediated metastatic ability in prostate cancer [27]. These results highlight the role of miR-23a in pancreatic cancer cell EMT. In contrast, numerous studies have shown that the expression of miR-23a is down-regulated and functioned as a tumour suppressor in osteosarcoma [18, 19]. These findings suggest that miR-23a expression and its biological functions may be tumour-type dependent.

Similar to classical transcription factors, miRNAs exert their effects by regulating specific target genes. It has been reported that miR-23a targets many important genes such as MTSS and IL-6R [15, 17]. In the present study, we compared our list of putative downstream genes of miR-23a (2681 genes) by the Affymetrix Gene Chip Human Gene 1.0 ST Array platform with a bioinformatics search in TargetScan 6.2 and identified 6 common genes (TGFB2, PIK3R3, FGF2, ESRP1, MAPK1 and FGFR3). In the present study, our results showed that only ESRP1 expression was significantly up-regulated in Panc-1 cells after transfecting with the miR-23a inhibitor (Supplementary Figure 2). Further investigation showed that ESRP1 was a direct functional target of miR-23a in pancreatic cancer. First, ESRP1 expression was up-regulated in epithelial phenotype cell lines (Aspc-1 and Bxpc-3), but down-regulated in mesenchymal cell lines (Cfpac-1 and Panc-1). ESRP1 expression was markedly down-regulated in the cells that underwent EMT. Second, dual-luciferase reporter assays showed that miR-23a directly targeted ESRP1 3'UTR. Third, miR-23a up-regulation decreased ESRP1 protein expression in Aspc-1 cells, while miR-23a down-regulation increased ESRP1 protein expression in Panc-1 cells. Four, ESRP1 mRNA was significantly decreased in pancreatic cancer tissues and lymph node metastases and was inversely correlated with miR-23a expression.

ESRP1 has been identified as a key regulator of splicing events related to EMT [28]. ESRP1 down-regulation is able to promote EMT in several solid cancers, such as pancreatic cancer and breast cancer [24, 29, 30], but further experiments will be necessary to clarify the conundrum of functions of miR-23a in inducing of pancreatic cancer cell EMT and metastasis by targeting ESRP1. In the present study, we found that ESRP1 up-regulation reversed the promotion of EMT and metastasis by miR-23a mimics in Aspc-1 cells. However, ESRP1 down-regulation rescued the miR-23a inhibitor-induced repression of EMT and metastasis in Panc-1 cells. Thus, the inhibition of ESRP1 expression by miR-23a is essential for pancreatic cancer cell EMT and metastasis.

CD44 is a multistructural and multifunctional cell surface molecule involved in cell proliferation, cell differentiation, cell migration, angiogenesis and cell survival. Alternative splicing of CD44 mRNA produces two protein isoform groups: the CD44 variants (CD44v) and the CD44 standard (CD44s). It has been shown that CD44s expression levels are associated with the mesenchymal phenotype in breast cancer [25, 31] and hepatocellular carcinoma [32], and has been implicated in promoting EMT, evidenced by decreased E-cadherin expression and increased N-cadherin and Vimentin expression [25]. Moreover, warzecha CC *et al.* [23] found that ESRP1 down-regulation promoted EMT and changes in FGFR2, CD44, CTNND1 (p120-Catenin) and ENAH transcripts. In pancreatic cancer, ESRP1 down-regulation promoted synthesis of the CD44s isoform, which further induces EMT [24]. In this study, we determined the regulatory relationship between miR-23a and ESRP1, and proposed that miR-23a may promote pancreatic cancer EMT and metastasis via regulating CD44 splice isoform switching. Thus, further study was needed to confirm the effect of miR-23a on CD44 splice isoform switching. Our results showed that miR-23a up-regulation inhibited the expression of ESRP1 and induced the switch from CD44v to CD44s in epithelial phenotype cells (Aspc-1). However, miR-23a down-regulation increased ESRP1 expression and reduced the switch from CD44v to CD44s in mesenchymal cells (Panc-1). Moreover, restoration of ESRP1 rescued the effect of miR-23a on CD44 splice isoform switching in pancreatic cancer cells. Therefore, miR-23a may affect CD44 splice isoform switching by directly regulating ESRP1, which consequently promoted EMT and metastasis.

In bladder and prostate cancers, there is a shift in the expression from FGFR2 IIIb to FGFR2 IIIc during EMT [33, 34]. In the present study, miR-23a up-regulation in Aspc-1 cells significantly decreased FGFR2 IIIb mRNA levels, and increased FGFR2 IIIc mRNA levels, but miR-23a down-regulation in Panc-1 cells leaded to opposite results. Restoration of ESRP1 rescued the effect of miR-23a on pancreatic cancer cells. In addition, Ueda J *et al.* [26] found that Panc-1 cells engineered to express ESRP1 exhibited increased FGFR-2 IIIb mRNA levels and decreased migration and invasion in PADC. However, Ueda J *et al.* [26] also found that ESRP1 up-regulation did not alter FGFR-2 IIIc mRNA levels. Perhaps this result is due to additional mechanisms that regulate FGFR-2 IIIc expression. Taken together, our results suggest that miR-23a partially promotes pancreatic cancer EMT and metastasis by targeting ESRP1 and regulating CD44 splicing as well as FGFR2 IIIb and FGFR2 IIIc mRNA levels (Figure 10E).

In summary, we identified a new mechanism by which miR-23a promotes pancreatic cancer cell EMT and metastasis by down-regulating ESRP1. These findings provide novel mechanistic insights into the role of miR-23a in EMT and metastasis.

## MATERIALS AND METHODS

### Patients and samples

A total of 52 pairs of human pancreatic cancer tissues and related cancer-adjacent normal tissues were obtained from patients who underwent surgical resection between January 2010 and August 2011 at the Southwest Hospital, Third Military Medical University. The follow-up date was ceased in December 2016. Another 10 primary pancreatic cancer samples with paired adjacent normal tissues and lymph node metastatic tissues were also obtained from the Southwest Hospital, Third Military Medical University. None of the patients had received chemotherapy or radiotherapy. This study was approved by the Ethics Committee of the Southwest Hospital, and informed consent was obtained from all the patients. The optimum cut-off value for the expression of miR-23a was selected using X-tile software version 3.6.1 (Yale University School of Medicine, USA) based on the association with the patients’ overall survival. The optimum cut-off value 3.5 was calculated by X-tile software based on the association with the patients’ overall survival. The miR-23a expression level more than or equal to 3.5 was regarded as high expression and less than 3.5 was regarded as low expression of miR-23a. The optimum cut-off value 3.7 was calculated by X-tile software based on the association with the patients’ disease free survival. The miR-23a expression level more than or equal to 3.7 was regarded as high expression and less than 3.7 was regarded as low expression of miR-23a (Supplementary Figure 1). The clinicopathological characteristics of the patients with pancreatic cancer were listed in Table 1.

### Cell lines and regents

Human pancreatic duct epithelial cells (PDC) and the pancreatic cancer cell lines Aspc-1, Bxpc-3, Cfpac-1, and Panc-1 were purchased from the cell bank at Shanghai Institutes for Biological Sciences (Chinese Academy of Sciences). PDC and Aspc-1 were cultured in RPMI-1640 medium (Invitrogen, CA, USA) containing 10% fetal bovine serum (Gibco, CA, USA). Bxpc-3, Cfpac-1, and Panc-1 cells were cultured in Dulbecco’s modified Eagle’s medium (Invitrogen, CA, USA) containing 10% FBS.

### EMT induction

Aspc-1 and Bxpc-3 cells were grown to approximately 60% confluency in either RPMI-1640 or DMEM with 10% FBS and then serum-starved overnight in RPMI-1640 or DMEM, respectively. TGF-β1 stimulation experiments were performed after cell treatment with recombinant human TGF-β1 (10 ng/ml; PeproTech, NJ, USA) for 72 h.

### Western blot analysis

Cells were harvested and lysed in RIPA buffer (Beyotime, Jiangsu, China), and the protein concentration was determined using a BCA Kit (Beyotime, Jiangsu, China). A 25 μg sample of protein was separated by sodium dodecyl sulfate polyacrylamide gel electrophoresis and then transferred to nitrocellulose membranes. The membranes were blocked with 5% non-fat powdered milk in PBS and then probed with primary antibodies. Rabbit monoclonal antibodies against E-cadherin, N-cadherin, Vimentin and β-actin were purchased from Proteintech (Wuhan, China); rabbit monoclonal antibodies against ESRP1 was purchased from Abcam (MA, USA); and mouse monoclonal antibody against CD44 was purchased from Cell Signaling Technology (MA, USA). After primary antibody incubation, the membranes were probed with HRP-conjugated secondary antibodies. The results were normalized to β-actin and expressed as relative density.

### RNA extraction and quantitative real-time PCR (qRT-PCR)

Total RNA from either cultured cells or tissue samples was extracted using a miRNeasy Mini Kit (QIAGEN, Duesseldorf, Germany), according to the manufacturer’s instructions. First-strand cDNA was synthesized using a miScriptII RT Kit (QIAGEN). The resulting cDNAs were used to analyse the expression of miR-23a, ESRP1, E-cadherin, N-cadherin, Vimentin, FOXO4, DYRK2, ESRP1, PDCD4, and TGFBR3 by qRT-PCR. And real-time PCR was performed using a miScript SYBR Green PCR Kit (QIAGEN) according to the manufacturer’s instructions. For ESRP1, E-cadherin, N-cadherin and Vimentin, FOXO4, DYRK2, ESRP1, PDCD4, and TGFBR3 mRNA expression, paired primers were used with β-actin as the endogenous control. For miR-23a expression, miRNA specific forward primer (miR-23a Fwd) and a universal reverse primer were used. U6 served as the endogenous control. The universal reverse quantitative PCR primer was provided in the miScript SYBR Green PCR Kit. All primers used were synthesized by Sangon Biotech (Shanghai, China) and were shown in Supplementary Table 1. All qRT-PCR assays were performed using a CFX96 Real-Time system (Bio-Rad, CA, USA). The qRT-PCR results were analysed using the 2^-ΔΔCt^ method. All experiments were performed in triplicate.

### Cell transfection

miR-23a mimics (miR-23a mimic) and its negative control (mimic NC), as well as miR-23a inhibitors (miR-23a inhibitor) and its negative control (inhibitor NC) were purchased from QIAGEN (Duesseldorf, Germany). The lentivirus vector for miR-23a down-regulation (miR-23a down) and miR-23a up-regulation (miR-23a up) and plasmid for ESRP1over-expression (ESRP1) were constructed by Sesh-biotech (Shanghai, China). ESRP1 siRNA was purchased from GenePharma (Shanghai, China). The sequences for the ESRP1 siRNA and the negative control (Con) used for the experiments were as follows:

ESRP1 siRNA: 5′-GCAGCAAGAUGGAACUUAUTT-3′, and

Con: 5′-UUCUCCGAACGUGUCACGUTT-3′.

Cells were seeded in 6-well or 96-well plates and transfected using Lipofectamine 2000 (Invitrogen, CA, USA) for 24 h or 48 h, according to the manufacturer’s protocol. Transfected cells were used for further analysis.

### Cell invasion assay

Aspc-1 cells were transfected with either miR-23a mimics alone or miR-23a mimics with the ESRP1 over-expression vectors for 48 h. Panc-1 cells were transfected with either miR-23a inhibitors alone or miR-23a inhibitors with ESRP1 siRNA for 48 h. For the cell invasion assay, transwell chambers were coated with 50 μg reconstituted basement membrane matrix (BD Biosciences, MD, USA). Then, 1 × 10^4^ cells in 100 μl serum-free medium were seeded into the upper chambers. Next, 600 μl of DMEM containing 5% FBS was added to the lower chamber. After 48 h of incubation, cells on the lower surface of the membrane were stained with crystal violet. The cells were counted in five random fields under a light microscope.

### Cell migration (wound healing) assay

Transfecte cells were seeded onto 6-well plates, grown to confluence, and starved for 24 h. Linear wounds were made by scraping the cells layer with a pipette tip. Cell motility was measured by photographing three random fields after 48 h.

### *In vivo* metastasis assay

Nude mice were provided by the Experimental Animal Centre of the Third Military Medical University. All animal studies were conducted in accordance with the Third Military Medical University animal use guidelines, and the protocols were approved by the Third Military Medical University Animal Care Committee. For the *in vivo* metastasis assay, 2×10^6^ Panc-1 cells infected with a miR-23a down-regulation lentivirus vector or a negative control lentivirus vector (NC) as well as 2×10^6^ Aspc-1 cells infected with a miR-23a up-regulation lentivirus vector or a negative control lentivirus vector (con) were suspended in 200 μl of phosphate-buffered saline (PBS) and injected into the splenic capsules of nude mice (ten per group, male nu/nu). After 6 weeks, the mice were sacrificed, their spleens and livers were dissected, the diameters of the primary splenic tumours were measured, and the mice that exhibited tumour metastasis were counted.

### Microarray analysis

Panc-1 cells were transfected with either the miR-23a inhibitor or inhibitor NC for 48 h. Total RNA was isolated using TRIzol reagent (Invitrogen, NY, USA). Synthesis of double-stranded cDNA, its conversion to target cRNA, and hybridization to the Affymetrix Gene Chip Human Gene 1.0 ST Array (Affymetrix, CA, USA) were performed according to the manufacturer’s instructions. The probe arrays were washed and stained using the Fluidics Station 450/250 and scanned using the Gene Chip Scanner 3000. Data analysis was conducted using Expression Console and Transcriptome Analysis Console v3.0 (Affymetrix, CA, USA). The genes with >2-fold differential expression between the miR-23a inhibitor group and the inhibitor NC group were submitted to the Biological Networks Gene Ontology tool (BiNGO,) for Gene Ontology (GO) analysis [22].

### Luciferase reporter assay

The wild-type ESRP1 3' UTR (1502 bp) containing the putative miR-23a binding sites (275-282) was amplified by PCR using genomic DNA as a template and then inserted into the downstream of the luciferase gene sequence in the psiCHECK-2 vector (Promega, WI, USA). The mutant-ESRP 3′UTR (deletion of AAAAUGU) was also accomplished by PCR. All vectors were identified by DNA sequencing. All primers used were synthesized by Sangon Biotech (Shanghai, China) and were shown in Supplementary Table 1.

Aspc-1 and Panc-1 cells were seeded into 96-well plates. For transfection, 2 μl of 20 μM of either miR-23a mimic or miR-23a inhibitor and 150 ng reporter plasmids (either wt-ESRP1 or mut-ESRP1) were mixed with 2 μl Lipofectamine 2000 (Invitrogen, CA, USA). Firefly luciferase activity was measured using a Dual-Luciferase Assay System (Promega, WI, USA) according to the manufacturer’s instructions and was normalized to *Renilla* activity.

### Reverse transcription–PCR

Total RNA was extracted from cultured cells, pancreatic cancer tissues and adjacent normal tissues with an RNeasy Mini Kit (QIAGEN GmbH, Hilden, Germany) according to the manufacturer’s instructions. cDNA was synthesized from 1 μg of total RNA using the SuperScript III First-Strand Synthesis SuperMix for reverse transcription (RT)–PCR (Invitrogen). All primers used were synthesized by Sangon Biotech (Shanghai, China) and were shown in Supplementary Table 1. The RT-PCR conditions for FGFR2-IIIb, FGFR2-IIIc and β-actin were as follows: 94°C for 2 min, 40 cycles at 94°C for 15 s, 58°C for 30 s, and 72°C for 30 s, with an extension step of 7 min at 72°C at the end of the last cycle.

### Statistical analysis

All statistical calculations were completed using SPSS 18.0 statistical software. Experimental data from 3 independent experiments were presented as the mean ± SD. The differences between groups were analysed using either Student’s t-test or one-way ANOVA. The Kaplan-Meier method was used for survival analysis and differences in survival were estimated using the Log-Rank test. *P* values < 0.05 were considered to be statistically significant.

## SUPPLEMENTARY MATERIALS FIGURES AND TABLES



## Abbreviations

EMT: epithelial-mesenchymal transition
ESRP1: Epithelial splicing regulatory protein 1
MET: mesenchymal-epithelial transition
miRNAs: MicroRNAs
3' UTR: 3'-untranslated region
PDAC: pancreatic ductal adenocarcinoma
BiNGO: Biological Networks Gene Ontology tool
GO: Gene Ontology
CD44v: CD44 variants
CD44s: CD44 standard
