# Supplementary material for: MicroRNA-23a promotes pancreatic cancer metastasis by targeting epithelial splicing regulator protein 1
Source: Oncotarget. 2017 Aug 24;8(47):82854–71. doi: 10.18632/oncotarget.20692 (PMC5669934; doi:10.18632/oncotarget.20692)
Supplement: Supplementary file 1 [file oncotarget-08-82854-s001.pdf]

MicroRNA-23a promotes pancreatic cancer metastasis by targeting epithelial splicing regulator protein 1

SUPPLEMENTARY MATERIALS

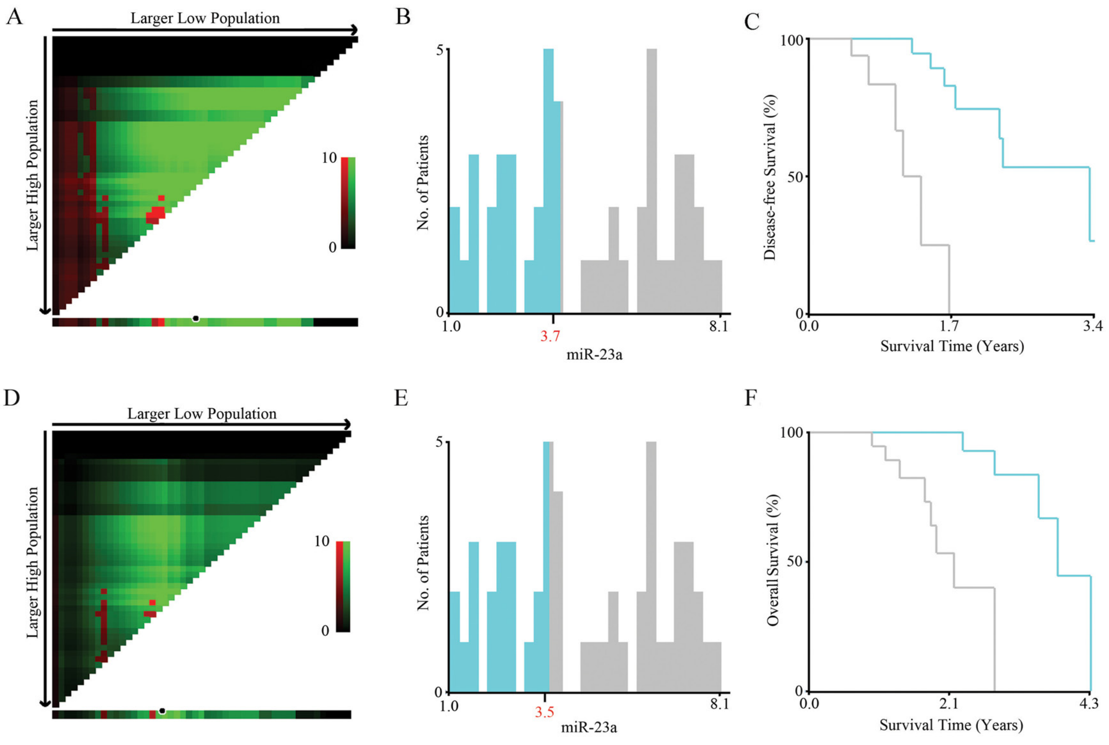

Supplementary Figure 1: X-tile analysis of survival data in pancreatic cancer patients reveals a continuous distribution based on miR-23a expression levels. The plot shows the χ^2 log-rank values produced when dividing the cohort with one cut-point, producing low and high subsets. The Y-axis represents cut-points from high to low (top to bottom) that defines a high subset, whereas the X-axis represents all potential cut-points from low to high (left to right) that defines a low subset. The optimal cut-point highlighted by the black circle in A and D is shown on a histogram of the entire cohort (B and E, for DFS and OS respectively), and a Kaplan-Meier plot (C and F, for DFS and OS respectively).

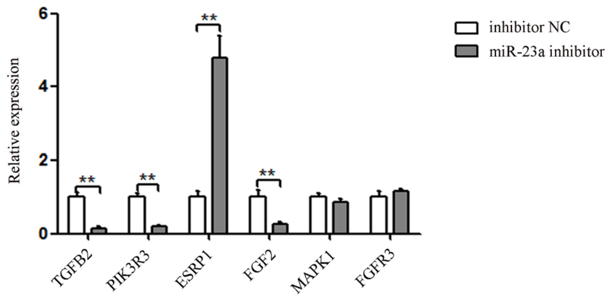

Supplementary Figure 2: miR-23a down-regulation increased the expression of ESRP1 mRNA level in Panc-1 cells after transfecting with the miR-23a inhibitor. The Panc-1 cells were transfected with miR-23a inhibitor or inhibitor NC. Next, the expression levels of TGFB2, PIK3R3, FGF2, ESRP1, MAPK1 and FGFR3 mRNA were determined by qRT-PCR.

Supplementary Table 1: The primers for PCR

Primer name	Primer sequence
miR-23a Fwd	5′- ATCACATTGCCAGGGATTTCC -3′
U6 Fwd	5′-TGCGGGTGCTCGCTTCGGCAGC -3′
ESRP1 Fwd	5′- CTGCTGTTCTGGAAAGTCGTGGAT-3′
ESRP1 Rev	5′- GGTTAAACTGTCGGAGGGCTTGG-3′
β-actin Fwd (qRT-PCR)	5′- CTCCATCCTGGCCTCGCTGT-3′
β-actin Rev (qRT-PCR)	5′- GCTGTCACCTTCACCGTTCC-3′
wt-ESRP1 3′UTR Fwd	5′ –AGGCGATCGCTCGAGAAGAAAACTTCTAGCAAATTCAGG -3′
wt-ESRP1 3′UTR Rev	5′ –ATTCCCGGGCTCGAGTTGAGATTAAACAGGCAAGTTTTAT -3′
mut-ESRP1 3′UTR-1	5′ - ACATCTGATTCCAAATTAAATGCCTATTTGCAATCAGC-3′
mut-ESRP1 3′UTR-2	5′- TTTGGAATCAGATGTCTCCATT -3′
FGFR2-IIIb Fwd	5′- GATAAATAGTTCCAATGCAGAAGTGCT -3′
FGFR2-IIIb Rev	5′- TGCCCTATATAATTGGAGACCTTACA -3′
FGFR2-IIIc Fwd	5′-GTGCTTGGCGGGTAATTCTA-3′
FGFR2-IIIc Rev	5′-GCTGAAGTCTGGCTTCTTGG-3′
E-cadherin Fwd	5′-A ACAGGATGGCTGAAGGTGA-3′
E-cadherin Rev	5′-CCTTCCATGACAGACCCCTT-3′
N-cadherin Fwd	5′-ATATTTCCATCCTGCGCGTG-3′
N-cadherin Rev	5′-GTTTGGCCTGGCGTTCTTTA-3′
Vimentin Fwd	5′-GAGAGGAAGCCGAAAACACC-3′
Vimentin Rev	5′-TTCCTGAATCTGAGCCTGCA-3′
FOXO4 Fwd	5′-TAGCACCACCTCCAGTCATG-3′
FOXO4 Rev	5′-AGTTGAAGTCCAGTCCCTCG-3′
DYRK2 Fwd	5′-ATTTCACCTTCCGCAACCAC-3′
DYRK2 Rev	5′-TCTCGGGCTTAAGGTCACAG-3′
PDCD4 Fwd	5′-TGAGATTTAAGGGCTGGGCA-3′
PDCD4 Rev	5′-ACCATCTCGACTCACTGCAA-3′
TGFBR3 Fwd	5′-CACTTTCCTATCCCGCAAGC-3′
TGFBR3 Rev	5′-TCAGGAGGCACACACTTAGG-3′
β-actin Fwd (RT-PCR)	5′-CTGGGACGACATGGAGAAAA-3′
β-actin Rev (RT-PCR)	5′-AAGGAAGGCTGGAAGAGTGC-3′

Supplementary Table 2: Gene ontology analysis of up- and down-regulated genes (related with proliferation and progression) (miR-23a inhibitor group vs. the inhibitor NC group)

GO terms representing molecular function

GO ID	Molecular function (GO description)	*P*-value	Count
GO:0046872	Metal ion binding	2.2985E-10	641
GO:0043167	Ion binding	3.5099E-10	647
GO:0043169	Cation binding	4.1596E-10	645
GO:0046914	Transition metal ion binding	6.0728E-8	400
GO:0008270	Zinc ion binding	9.3019E-7	350
GO:0003824	Catalytic activity	4.6267E-6	795
GO:0003779	Actin binding	2.3880E-5	72
GO:0003707	Steroid hormone receptor activity	7.3615E-5	18
GO:0016616	Oxidoreductase activity, acting on the CH-OH group of donors, NAD or NADP as acceptor	1.3530E-4	29
GO:0008092	Cytoskeletal protein binding	1.4159E-4	100
GO:0004879	Ligand-dependent nuclear receptor activity	1.7433E-4	18
GO:0005536	Glucose binding	2.7339E-4	5
GO:0004030	Aldehyde dehydrogenase [NAD (P)+] activity	2.7339E-4	5
GO:0055103	Ligase regulator activity	3.7146E-4	4
GO:0055106	Ubiquitin-protein ligase regulator activity	3.7146E-4	4
GO:0048037	Cofactor binding	4.0495E-4	55
GO:0000267	Cell fraction	1.7844E-4	186
GO:0012505	endomembrane	2.4886E-4	238
GO:0043408	Regulation of MAPKKK cascade	1.6900E-4	40
GO:0042175	Nuclear membrane-endoplasmic reticulum network	2.7778E-4	115
GO:0044432	Endoplasmic reticulum part	2.9438E-4	123
GO:0031226	Intrinsic to plasma membrane	3.0069E-4	202
GO:0071212	Subsynaptic reticulum	3.4992E-4	128
GO:0016323	Basolateral plasma membrane	4.2999E-4	46
GO:0005792	Microsome	4.3812E-4	52
GO:0042598	Vesicular fraction	4.9120E-4	53
GO:0005887	Integral to plasma membrane	5.2630E-4	197
GO:0005913	Cell-cell adherens junction	5.3520E-4	13
GO:0045121	Membrane raft	6.3833E-4	35
GO:0005634	nucleus	6.7338E-4	765

GO terms representing cellular compartment

GO ID	Cellular component (GO description)	*P*-value	Count
GO:0044424	Intracellular part	4.4221E-14	1624
GO:0044464	Cell part	4.3331E-10	2091
GO:0005737	Cytoplasm	1.8983E-9	1158
GO:0043229	Intracellular organelle	1.2372E-8	1374
GO:0043231	Intracellular membrane-bounded organelle	3.3832E-8	1241
GO:0045177	Apical part of cell	1.6175E-7	56
GO:0004444	Cytoplasmic part	1.1638E-6	790
GO:0044459	Plasma membrane part	1.6401E-6	338
GO:0070161	Anchoring junction	1.5612E-5	151
GO:0016328	Lateral plasma membrane	6.8643E-5	9
GO:0005912	Adherens junction	7.0687E-5	35
GO:0005624	Membrane fraction	7.6517E-6	145
GO:0016324	Apical plasma membrane	1.0118E-4	40
GO:0005911	Cell-cell junction	1.0917E-4	45
GO:0005626	Insoluble fraction	1.5401E-4	148
GO:0005789	Endoplasmic reticulum membrane	1.5702E-4	114
GO:0005783	Endoplasmic reticulum	1.7164E-4	177
GO:0000267	Cell fraction	1.7844E-4	186
GO:0012505	endomembrane	2.4886E-4	238
GO:0043408	Regulation of MAPKKK cascade	1.6900E-4	40
GO:0042175	Nuclear membrane-endoplasmic reticulum network	2.7778E-4	115
GO:0044432	Endoplasmic reticulum part	2.9438E-4	123
GO:0031226	Intrinsic to plasma membrane	3.0069E-4	202
GO:0071212	Subsynaptic reticulum	3.4992E-4	128
GO:0016323	Basolateral plasma membrane	4.2999E-4	46
GO:0005792	Microsome	4.3812E-4	52
GO:0042598	Vesicular fraction	4.9120E-4	53
GO:0005887	Integral to plasma membrane	5.2630E-4	197
GO:0005913	Cell-cell adherens junction	5.3520E-4	13
GO:0045121	Membrane raft	6.3833E-4	35
GO:0005634	nucleus	6.7338E-4	765
